# Effects of Orange Peel-Derived Carbon Sources on *Nannochloropsis salina* Mixotrophic Cultivation

**DOI:** 10.3390/biotech15030065

**Published:** 2026-08-07

**Authors:** Carlo Esposito, Giancarlo Aldini, Yanan Yin, Stefano Gandolfi, Gianluca Ottolina, Francesco Secundo

**Affiliations:** 1 Istituto di Scienze e Tecnologie Chimiche “Giulio Natta”, Consiglio Nazionale delle Ricerche (CNR), Via Mario Bianco 9, 20131 Milano, Italy; carlo.esposito@unimi.it (C.E.);; 2 Department of Pharmaceutical Sciences, University of Milan, Via Luigi Mangiagalli, 25, 20133 Milano, Italy; 3 Institute of Nuclear and New Energy Technology (INET), Tsinghua University, Beijing 100084, China

**Keywords:** orange peel, fatty acids, antioxidants, eicosapentaenoic acid, *Nannochloropsis salina*

## Abstract

Microalgae are recognized as sustainable biofactories for metabolites relevant to nutraceutical, pharmaceutical, and marine drug applications. *Nannochloropsis salina* is notable for its production of bioactive lipids, including the pharmaceutically relevant omega-3 eicosapentaenoic acid (EPA). Here, we evaluated orange peel extract (OPE), a citrus by-product, as a substrate for mixotrophic cultivation of *N. salina* within a bioeconomy framework. OPE supplementation triggered dose-dependent physiological responses. Among the tested OPE concentrations, 5% supplementation resulted in the highest total fatty acid content, increasing fatty acids from 24.4% (control) to 30.6% and EPA from 6.4% to 9.8%, whereas 10% OPE maintained biomass pigmentation. Higher OPE levels enhanced antioxidant potential, raising total antioxidant capacity from 4.6 (control) to 6.7 mg/g vitamin C equivalents (with 10% OPE), while the highest concentrations induced metabolic stress, reducing biomass and altering lipid composition. Fourier transform infrared spectroscopy analyses revealed biochemical adjustments consistent with metabolic reorganization, including increased intensities of the amide I and II bands associated with protein-rich structures. Overall, OPE emerges as a cost-effective supplement capable of modulating the biochemical quality of *N. salina* while valorizing agro-industrial residues. The increases in EPA and antioxidant capacity support the potential of OPE-supplemented cultures as a platform for the production of marine-derived bioactive compounds.

## 1. Introduction

Microalgae are emerging as sustainable biofactories for high-value metabolites, including proteins, lipids, vitamins and pigments, with applications in the nutraceutical, pharmaceutical, cosmetic and feed industries [1,2].

In this context, the marine microalga *Nannochloropsis salina* is of particular industrial interest due to its high content of polyunsaturated fatty acids (PUFAs), especially eicosapentaenoic acid (EPA), making it a promising alternative to fish oil and a valuable ingredient for pharmaceutical applications, as oils extracted from *Nannochloropsis* sp. have been shown to reduce total cholesterol by up to 25% in treated patients [3,4,5]. In a balanced diet, the primary sources of omega-3 fatty acids are traditionally associated with fish consumption or the use of dietary supplements. Furthermore, *Nannochloropsis* sp., due to its high EPA content, represents a promising sustainable alternative to meet the requirement for these essential fatty acids [5].

Beyond its PUFA content, *N. salina* also produces other valuable compounds, including β-carotene, several xanthophylls (violaxanthin, vaucheriaxanthin, canthaxanthin, astaxanthin, neoxanthin, zeaxanthin, and antheraxanthin), and phenolic compounds [6]. These bioactive compounds find applications in the food, cosmetic, and health-care industries [7]. Notably, the carotenoids from *N. salina* show antioxidant and anti-inflammatory activities [8,9], as well as antiproliferative effects against human cancer cells [10]. Its broad ecological distribution and tolerance to fluctuating environmental conditions further enhance its biotechnological potential [5,11,12]. A major obstacle to large-scale cultivation, however, is the cost of nutrient inputs. Agro-industrial residues, particularly citrus-processing by-products, represent an abundant, low-cost and nutrient-rich carbon source that can simultaneously reduce production costs and environmental impact [13]. Orange peel, which can account for up to 60% of the processed fruit mass [14,15], contains fermentable sugars, organic acids and micronutrients capable of supporting algal growth within a circular bioeconomy framework.

These valuable components can be recovered through extraction processes, such as aqueous or solvent-based extraction, yielding orange peel extract (OPE), which represents a promising strategy for the valorization of this agro-industrial by-product. Although OPE has been shown to stimulate lipid accumulation in microalgae such as *Chlorella vulgaris* [14,16], its effects on *N. salina* remain poorly characterized. This study investigates the use of OPE as a renewable organic substrate for the cultivation of *N. salina*, focusing on biomass productivity, lipid yield, fatty acid composition, and the accumulation of antioxidants and phenolic metabolites. This approach exemplifies a closed-loop biorefinery concept in which agricultural residues are biologically converted into high-value algal biomass for applications in the food, cosmetic and pharmaceutical sectors [17,18].

## 2. Materials and Methods

### 2.1. Material Preparation

The strain *N. salina* (CCMP 369) was provided by the Institute of Biomolecular Chemistry of the National Research Council, Naples, Italy. An initial microalgal culture was established by inoculating 200 mL of *N. salina* culture into a 500 mL flask containing f/2 Guillard growth medium, according to the procedure described by Guillard [19]. Briefly (NaNO_3_ (75 mg L^−1^), NaH_2_PO_4_·H_2_O (5 mg L^−1^). FeCl_3_·6H_2_O (3.15 mg L^−1^), Na_2_EDTA·2H_2_O (4.36 mg L^−1^), CuSO_4_·5H_2_O (0.01 mg L^−1^), ZnSO_4_·7H_2_O (0.022 mg L^−1^), CoCl_2_·6H_2_O (0.01 mg L^−1^), MnCl_2_·4H_2_O (0.18 mg L^−1^) e Na_2_MoO_4_·2H_2_O (0.006 mg L^−1^) (Merck; Darmstadt, Germany). Vitamins were added to reach final concentrations of thiamine HCl (100 µg L^−1^), biotin (0.5 µg L^−1^), and cyanocobalamin (1 µg L^−1^). When the culture reached an optical density (OD_680_) of 1.01 (exponential phase), the culture was used for experiments. The inoculum cultures were maintained at 25 ± 1 °C (Eppendorf Innova 42 New Brunswick) operating at a constant mixing speed of 120 rpm throughout the entire cultivation period, including the dark phase, and equipped with a photosynthetic lamp. Illumination followed a circadian cycle of 14 h of light and 10 h of darkness with an intensity of 24 µmol m^−2^ s^−1^ (emission spectrum: 360–760 nm; peaks at 450 nm and 680 nm).

### 2.2. Supplement and Medium Preparation

Orange peel waste (OPW) of Tarocco variety (Sicily, Italy), a by-product of juice industrial production, was used to prepare the feed supplement for microalgae. In particular, 1.36 kg of OPW was crushed with a manual shredder, mixed with water (2 L) and stirred overnight. The resulting mixture was filtered through Whatman No. 1 paper and centrifuged (5000 rpm, 1 h), and the liquid fraction (OPE) was used directly as a supplement without further treatment. Growth media were prepared by adding OPE (containing 23 g/L of glucose equivalent, see Section 2.11 for glucose equivalent assay) at different concentrations (5%, 10% and 15%). To prepare 1 L of medium, the appropriate amount of OPE was added to 22 micron filtered seawater, which was then sterilized. After cooling, the necessary components were added to obtain the complete f/2 medium.

### 2.3. Culture Experiment Preparation

Cultures were prepared in 100 mL flasks with a final volume of 50 mL. *N. salina* grown in standard f/2 medium was used as the control. For the experimental conditions, f/2 medium supplemented with different concentrations of OPE was used, and the microalgae were subsequently added to reach a final optical density (OD) of 0.1. All cultures were maintained under the conditions previously described for 288 h, with the addition of the antibiotics ampicillin (100 µg/mL) and kanamycin (20 µg/mL).

### 2.4. Growth Measurement of N. salina

Samples were centrifuged and resuspended in the same amount of milliQ water before analysis. The cell growth of *N. salina* was monitored by measuring both OD (optical density) at 680 nm using a spectrophotometer (Jasco V-730, JASCO Corporation, Hachioji, Tokyo, Japan) and chlorophyll a fluorescence recorded by a Jasco FP-8350 equipped with a Jasco FMP-125 spectrofluorometer (JASCO Corporation). The measurements were carried out using an excitation wavelength of 435 nm. Emission spectra were acquired in the 400–700 nm range with a spectral step of 0.5 nm, following the procedure described [20]. Prior to analysis, samples were diluted twofold (1:2) or adjusted to an optical density (OD) within the range of 0.1–0.8 to minimize possible effects related to high cell concentration, including detector saturation and fluorescence quenching. The chlorophyll a fluorescence signal was evaluated by monitoring the emission maximum at 680 nm. Spectral corrections were performed by subtracting the fluorescence background of the corresponding blanks, consisting of f/2 medium and f/2 medium containing OPE.

### 2.5. Growth Rate Determination

The specific growth rate of each culture was calculated from the slope of the linear regression of time and natural logarithm optical density in the exponential growth phase:

(1) $$µ=\frac{\left(\ln N-\ln N_{0}\right)}{\left(t-\;t_{0}\right)}$$

where µ (d^−1^) is the specific growth rate in the exponential growth phase, *N*_0_ is the optical density at the beginning of the exponential phase (*t*_0_), and *N* represents the optical density at time t (expressed in days) of the exponential phase.

### 2.6. Recovery of Biomass from Cell Cultivation

The biomass was transferred to 50 mL centrifuge tubes and centrifuged at 10,000× *g* for 5 min. The supernatant was removed, and the pelletized biomass was washed with 40 mL MilliQ water three times. After centrifugation, the residue was freeze-dried.

### 2.7. Determination of Orthophosphate (PO_4_^3−^)

Phosphate concentrations in the culture medium were measured using the Merck Phosphate Test (no. 1.14848.0001) according to the manufacturer’s instructions. For quantification, a calibration curve was prepared from KH_2_PO_4_ standard solutions obtained by serial dilutions from a 1.475 mg/L stock solution (*n* = 6, range 0.006–1.475 mg/L, coefficient of determination (R^2^) = 0.9998, y = 0.763x + 0.0006), where y is Abosorbance and x is mg/L PO_4_^3−^.

### 2.8. Fatty Acid Extraction and Gas Chromatography with Flame Ionization Detection (GC-FID) Analysis

The fatty acid composition was determined according to Kumar et al. [21]. Briefly, 3 mg of freeze-dried biomass was subjected to lipid transesterification by adding 0.3 mL of 0.35 M KOH (Sigma-Aldrich, Steinheim, Germany) dissolved in methanol (Honeywell, Seelze, Germany). The samples were sonicated for 10 min in a bath sonicator operating at 30 kHz and subsequently incubated at 60 °C with shaking at 400 rpm for 25 min in a thermo-shaker.

After incubation, 0.3 mL of 0.64 M H_2_SO_4_ (Sigma-Aldrich, Germany) in methanol was added to promote the conversion of fatty acids into their corresponding fatty acid methyl esters (FAMEs). The reaction mixture was cooled on ice for 5 min, and the obtained FAMEs were extracted three consecutive times using 1 mL of petroleum ether containing nonadecanoic acid (C19:0) as an internal reference compound.

FAME analysis was performed by gas chromatography coupled with flame ionization detection (GC-FID) using an Agilent 6890 GC system equipped with a MEGA-WAX capillary column (25 m × 320 µm × 0.15 µm; MEGA, Legnano, Italy). Hydrogen was employed as the carrier gas under a constant pressure of 30 kPa. The oven temperature was initially set at 60 °C and maintained for 2 min, then increased to 150 °C at a rate of 15 °C/min, followed by an increase to 195 °C at 3 °C/min and finally to 220 °C at 5 °C/min. Fatty acid identification was achieved by comparing retention times with those obtained from a commercial 37-component FAME standard mixture (Sigma-Aldrich, Steinheim, Germany).

### 2.9. Antioxidant Activity and Total Polyphenol Content

#### 2.9.1. Extract Preparation

Extraction was performed by modifying the procedure implemented by Barone et al. [22]. Briefly, 0.5 mL of methanol was added to 2 mg of lyophilized biomass, and the mixture was vortexed for 30 s at 3000 rpm, followed by sonication in an ultrasonic bath for 10 min. The samples were then incubated in the dark at room temperature for 20 min before centrifugation at 1500× *g* for 5 min. The supernatant was collected, and the remaining biomass pellet was processed again under the same conditions. The resulting supernatants were combined to a final volume of 1 mL and stored at –20 °C until further analysis.

#### 2.9.2. Folin–Ciocalteu Assay

The total phenolic content, expressed as gallic acid equivalents (GAE), was determined using the Folin–Ciocalteu assay according to Conlon et al. [23]. Briefly, 0.1 mL of sample extract was mixed with 0.5 mL of Folin–Ciocalteu reagent (VWR Chemicals, Milano, Italy) diluted 1:10. After 5 min, 0.4 mL of saturated sodium carbonate solution (37.5 g/L) was added, and the mixture was incubated in the dark at room temperature for 40 min. Absorbance was measured at 760 nm using a UV–Vis spectrophotometer (Jasco V-730). GAE values were calculated from a gallic acid calibration curve (*n* = 5, range 15–125 μg/mL, R^2^ = 0.9988, y = 0.0098x + 0.0091), where y is Absorbance and x is gallic acid concentration (μg/mL).

#### 2.9.3. DPPH Free Radical Scavenging Assay

The assay was performed following the procedure of Conlon [23] with some modifications. A 0.25 mM 2,2-diphenyl-1-picryl-hydrazyl (DPPH) (Tokyo Chemical Industry, Tokyo, Japan) solution was diluted with methanol: water (50% *v*/*v*) to obtain an absorbance of 0.70 ± 0.05 at 515 nm. For analysis, 0.9 mL of the diluted DPPH solution was added to 0.1 mL of sample extract. After incubation in the dark for 30 min at room temperature, the absorbance of samples and controls was measured at a wavelength of 515 nm using a ultraviolet–visible (UV–Vis) spectrophotometer (Jasco V-730). Vitamin C equivalent values were calculated using a vitamin C calibration curve (*n* = 6, range 4.7–150 μM, R^2^ = 0.9934, y= −0.0022x + 0.6633), where y is Absorbance and x is vitamin C concentration (μM/mL).

### 2.10. Pigment Analysis

Chlorophyll and total carotenoid contents were determined spectrophotometrically following the methods of Maadane et al. [24] and Lichtenstein and Buschmann [25]. The absorbance (A) of the pigment extracts was measured at 470, 648, and 664 nm using methanol as a blank. In the equations, the subscripts of A (e.g., A_470_, A_648_, and A_664_) indicate the wavelength (nm) at which the absorbance was measured. Pigment concentrations (chlorophyll *a*, chlorophyll *b*, and total carotenoids) were calculated according to the Lichtenthaler equations (Chl *a*: chlorophyll *a*; Chl *b*: chlorophyll *b*).

(2) $$\mathrm{Chl}\;a\;\left({\mu g\;\mathrm{mL}}^{-1}\right)=\left(16.72\times {\;A}_{664\;}\right)-\left(9.16\times {\;A}_{648\;}\right)$$

(3) $$\mathrm{Chl}\;b\;\left({\mu g\;\mathrm{mL}}^{-1}\right)=\left(34.09\times {\;A}_{648\;}\right)-\left(15.28\times {\;A}_{664\;}\right)$$

(4) $$Carotenoids_{total}\;\left({\mu g\;\mathrm{mL}}^{-1}\right)=\frac{\left(1000\times {\;A}_{470\;}\right)-\left(1.63\times \mathrm{Chla}\right)-\left(104.96\times \;\mathrm{Chlb}\right)}{221}$$

### 2.11. DNS Assay

The concentration of reducing sugars was determined by the 3,5-dinitrosalicylic acid (DNS) method [26]. DNS reagent (Thermo Scientific, Waltham, MA, USA) was prepared by dissolving 0.25 g of DNS in 12.5 mL of Milli-Q water, adding 7.5 g of sodium-potassium tartrate tetrahydrate and 5 mL of 2 M NaOH, then diluting to a final volume of 25 mL.

Samples were obtained by taking 1 mL of culture and centrifuging at 12,000× *g* for 5 min; the supernatant was then removed and placed in a 2 mL test tube.

Afterward, 330 µL of DNS reagent solution was added. The mixture was heated to 98 °C for 5 min with a thermoshaker (500 rpm), cooled on ice, and centrifuged at 12,000× *g* for 3 min. Subsequently, a 200 µL aliquot was further diluted with Milli-Q water to 1 mL, and the absorbance was measured at 540 nm. For quantification, a calibration curve obtained with a glucose standard solution prepared by serial dilutions from a 2.5 mg/mL stock solution was used (*n* = 5, range 0.04–2.5 mg/mL, R^2^ = 0.9972, y = 0.1567x + 0.0119), where y is absorbance, and x is glucose concentration (mg/mL).

### 2.12. FT-IR Spectroscopy

A lyophilized sample of approximately 0.5–1 mg of each culture was ground with 250 mg of KBr and then dried in an oven for 12 h. After the samples were completely dry, the pellet was prepared by means of a hydraulic press and then analyzed. Spectra were collected in the wavenumber range between 400 and 4000 cm^−1^. The collected spectra were baseline corrected using the Jasco Spectra Manager software (version 1.30.00). Fourier transform infrared spectroscopy (FT-IR) spectra were recorded using an FT/IR-610 Jasco instrument (JASCO Corporation) as the average of 32 scans at 2 cm^−1^ resolution. The collected spectra were baseline-corrected, and peak areas were determined using the Jasco Spectra Analysis software (version 1.30.00).

### 2.13. Data Treatment

Results are reported as mean ± standard deviation (SD). The effects of species and experimental conditions were evaluated through one-way ANOVA, and significant differences were further examined using Tukey’s post hoc test (*p* = 0.05). Relationships between the different variables were explored by calculating Pearson correlation coefficients. Statistical analyses were carried out with Jamovi software (v2.3.28.0).

## 3. Results and Discussion

### 3.1. Microalgal Cultivation in OPE Medium

The OPW from the agro-industrial supply chain is rich in components that can be used to support microalgal growth. In particular, aqueous extraction of orange peels enabled the recovery of a supplement enriched in sugars, containing approximately 23 g/L of glucose equivalents and a phosphate concentration of about 70 mg/L. Generally, the sugar profile of OPE is characterized by a sucrose/glucose/fructose mass ratio of approximately 1.0/3.3/5.8 [27]. However, beyond the extraction parameters (e.g., temperature, extraction medium, time of infusion), the amount of soluble sugars extracted can also vary depending on the specific composition of the peels [14,28]. The measured phosphate concentrations were lower than those reported in previous studies [14]. To evaluate the effect of the organic supplement, OPE, it was added to the standard f/2 medium at final concentrations of 5%, 10%, and 15% (*v*/*v*) (Table 1). The amount of supplement added is a key parameter, as its high carotenoid content can color the medium, potentially reducing light penetration and affecting the photosynthetic performance of the microalgae. The extraction of orange peel components was performed using Milli-Q water to selectively solubilize the sugar fraction while minimizing the co-extraction of hydrophobic or potentially inhibitory compounds, such as limonene, which could impair microalgal growth. Nevertheless, OPE is a complex mixture containing organic acids, phenolic compounds, and micronutrients. These molecules may exert beneficial effects by providing nutrients or stimulating metabolic pathways, but they may also interfere with algal metabolism depending on their concentration. The use of water also facilitates subsequent sterilization steps and prevents the introduction of undefined organic molecules that may degrade into toxic species during medium preparation. However, excessive supplementation can reduce the salinity of the f/2 medium, generating osmotic imbalances unfavorable to microalgal physiology. Limiting the supplementation to a maximum of 15% enables assessment of the nutritional contribution of OPE without significantly altering key physicochemical parameters, particularly salinity. *N. salina* is known to tolerate a wide salinity range, far broader than the minor variations introduced by OPE addition, and fluctuations of up to 15% do not significantly hinder its growth [29]. Although OPE is not a significant nitrogen source (<1 mg L^−1^ total nitrogen) [14,30], no additional nitrogen was supplied to adjust the optimal nitrogen (N)-to-phosphorus (P) ratio (N:P ratio), as *N. salina* tolerates nitrogen-limited conditions. This approach allowed growth to be evaluated under conditions that better reflect those encountered in its natural environment. While growth could be enhanced by providing excess nutrients and CO_2_ [31], maintaining nitrogen limitation offers a more ecologically relevant framework for interpretation.

### 3.2. Growth Measurement

**Figure 1:**
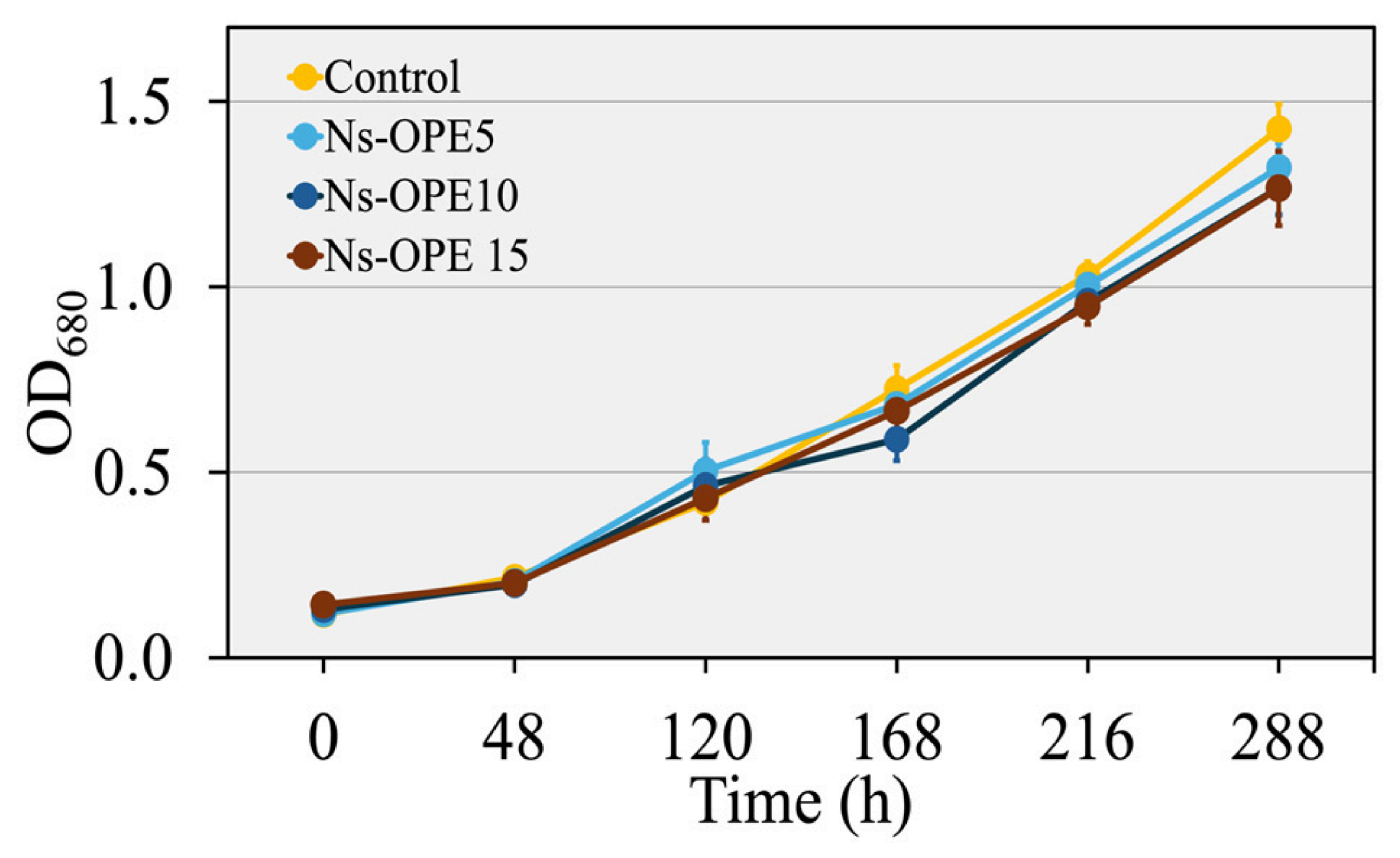
Microalgae growth monitored by recording absorbance at 680 nm (OD_680_) over time (0–288 h) for cultures of *N. salina*. Values are means ± standard deviation (SD), *n* = 3.

The growth of *N. salina* was determined using OD_680_ nm. Additionally, physical parameters such as pH and sugar consumption were measured. Optical density analysis (Figure 1) revealed a steady growth of *N. salina* cultures during the entire experiment. The results showed that the cultures treated with OPE exhibited maximum specific growth rates (Equation (1)) similar to the control (0.30 ± 0.03 d^−1^). The µ_max_ values of the OPE treatments ranged from 0.25 ± 0.04 d^−1^ (*Ns*-OPE 15%) to 0.30 ± 0.06 d^−1^ (*Ns*-OPE 5%), with no statistically significant differences among treatments. These values are consistent with the literature, which reports µ_max_ for *Nannochloropsis* ranging between 0.11 and 0.21 d^−1^ [32].

**Figure 2:**
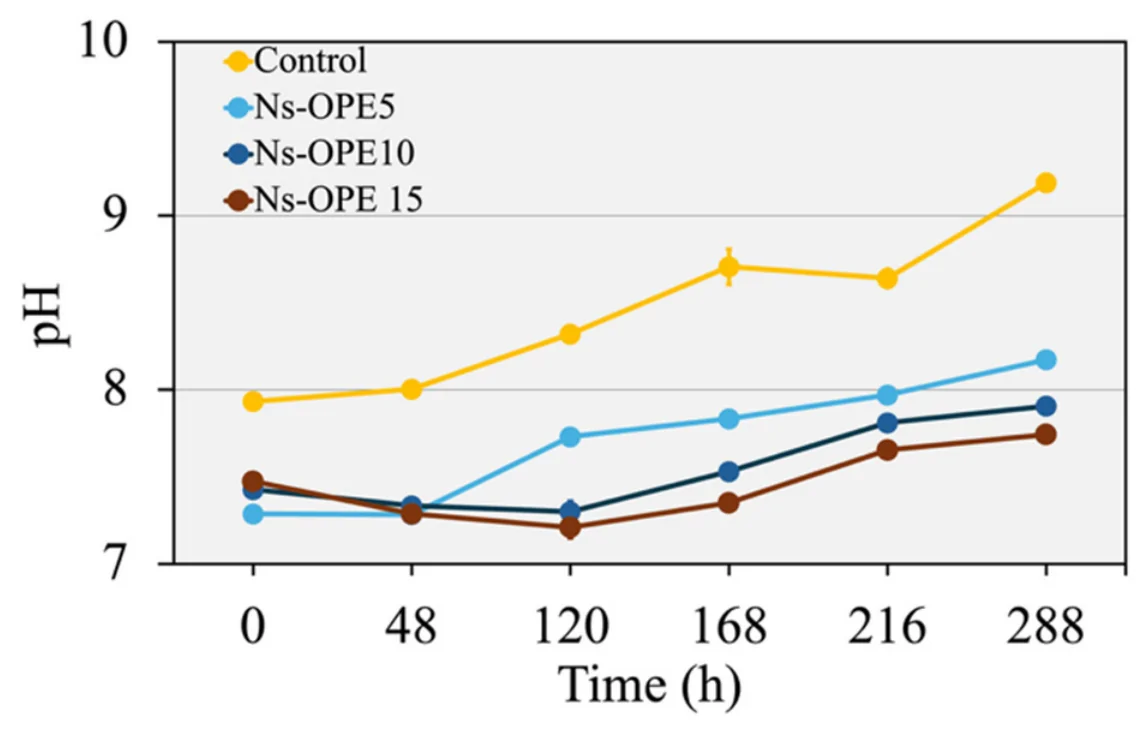
pH variation at different times (0–288 h) of culture of *N. salina* with different OPE concentrations. Values are means ± SD, *n* = 3.

The results in Figure 2 show the pH variation in *N. salina* cultivated in f/2 medium supplemented with different concentrations of OPE. The OPE supplement has an acidic pH of approximately 4.5; consequently, the f/2 medium with OPE was then adjusted to pH 7.5 with NaOH before sterilization. *N. salina* grows over a broad pH range (5.0–10.5) and exhibits an optimum between 7.5 and 8.5 [33,34]. Throughout cultivation, all cultures exhibited a steady upward shift in pH, converging toward the optimal range of 7.5–8.2 in the OPE-supplemented cultures and around 9.2 in the control culture. The *Ns*-OPE10 and *Ns*-OPE15 cultures showed an early pH decline during the first 120 h, indicating a transient metabolic adjustment to the growth conditions, whereas *Ns*-OPE5 and the control cultures did not exhibit this initial acidification. The subsequent pH rise in all treatments is largely attributable to photosynthetic activity, which removes protons from the medium [35].

### 3.3. Biomass Accumulation, Chlorophyll and Sugar Utilization

The addition of OPE to the culture medium resulted in a significant reduction in cellular dry weight (Table 2). Although OD measurements indicated similar growth among treatments, biomass dry weight analysis showed a reduction of approximately 30% compared to the control (*p* < 0.001). Although the OD values remained comparable across treatments, the lower final dry biomass in OPE-supplemented cultures may be linked to the phosphate content introduced with the extract. Under the conditions tested, OPE supplementation altered both biomass composition and overall productivity. Because OPE also changed the N:P ratio of the culture medium, the physiological responses observed cannot be attributed solely to mixotrophy; phosphorus availability likely played a substantial role. Thus, the effects measured arise from the combined influence of these factors rather than from organic carbon addition alone. Moreover, previous studies have shown that in *N. salina*, high phosphate concentrations (>5 mg L^−1^) can increase cell size without increasing cell number, due to intracellular accumulation of polyphosphate granules [36].

Fluorescence spectroscopy enables the monitoring of various classes of biomolecules, including chlorophylls, which are abundant in microalgae [20,37]. *N. salina*, like other species of the genus, contains only chlorophyll *a* [38], which exhibits emission between 640 and 670 nm after excitation in the 350–450 nm range. Chlorophyll (Chl) levels at 288 h (Table 2) were significantly higher with 10% OPE (*p* < 0.05) but declined at 15% OPE.

**Figure 3:**
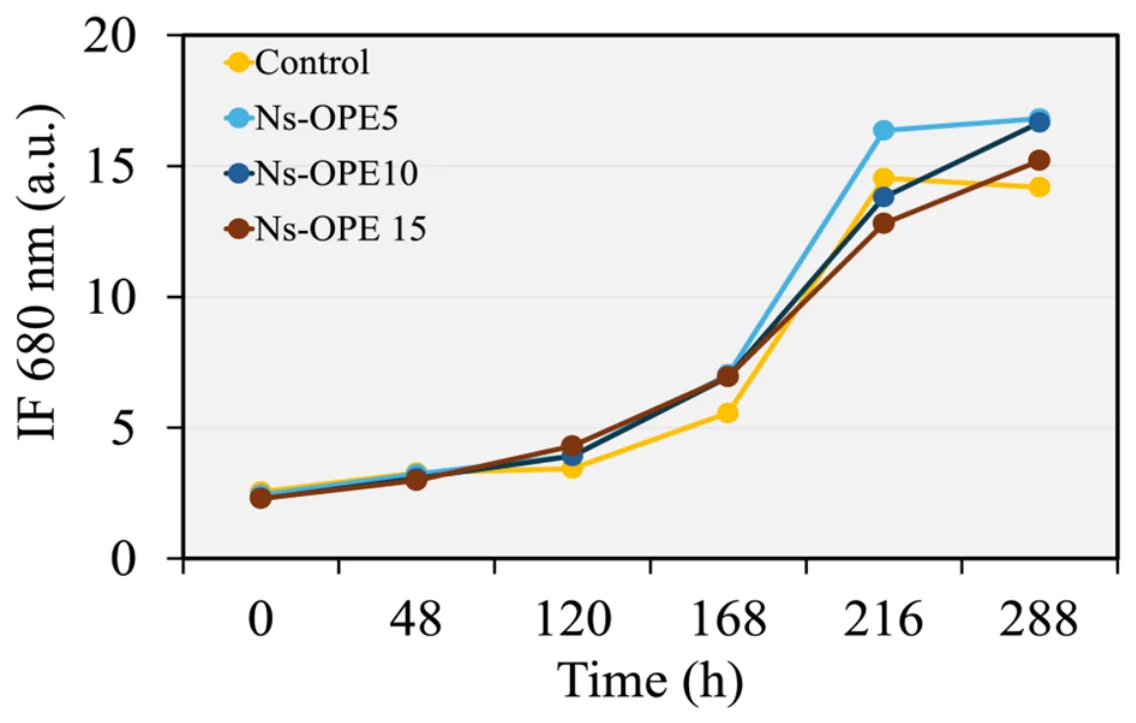
Variation in fluorescent intensity (IF) of *N. salina* at different concentrations of OPE. Excitation wavelength 435 nm. Values are means ± SD, *n* = 3.

These trends were consistent with fluorescence measurements (Figure 3), which showed an enhanced signal after 288 h in the 10% OPE samples. Despite nitrogen being a potential limiting factor, *Nannochloropsis* maintains the ability to perform photosynthesis under nitrogen-depleted conditions through extensive remodeling of the photosynthetic apparatus. This includes changes in pigment composition and a coordinated reduction in all major electron transport complexes, such as photosystem II, photosystem I, and the cytochrome b6f complex [31]. Such adaptive mechanisms may contribute to the pigment modulation observed in our cultures, particularly under OPE supplementation, which may further limit light penetration. The level of carotenoids with OPE addition was comparable to the control. In the *Nannochloropsis* genus, the most abundant carotenoids are violaxanthin, vaucheriaxanthin, and β-carotene, while others such as neoxanthin, antheraxanthin, zeaxanthin, canthaxanthin, and astaxanthin typically accumulate only in trace amounts [39]. However, carotenoid content is highly responsive to environmental and nutritional factors [40]. The concentrations measured in this study are consistent with those previously reported for *N. salina* [41]. Carotenoid biosynthesis is tightly regulated [42] and may proceed through distinct metabolic routes [43]. In *Nannochloropsis*, increases in carotenoids are often triggered by stress or restrictive conditions, including reduced light penetration due to OPE coloration, or shifts in nitrogen, phosphorus, and sulfur availability [7]. Accordingly, Pearson’s correlation analysis (Table S1) revealed a strong positive relationship between chlorophyll a and total carotenoids (r = 0.814; *p* < 0.001), suggesting a coordinated pigment response under the experimental conditions.

**Figure 4:**
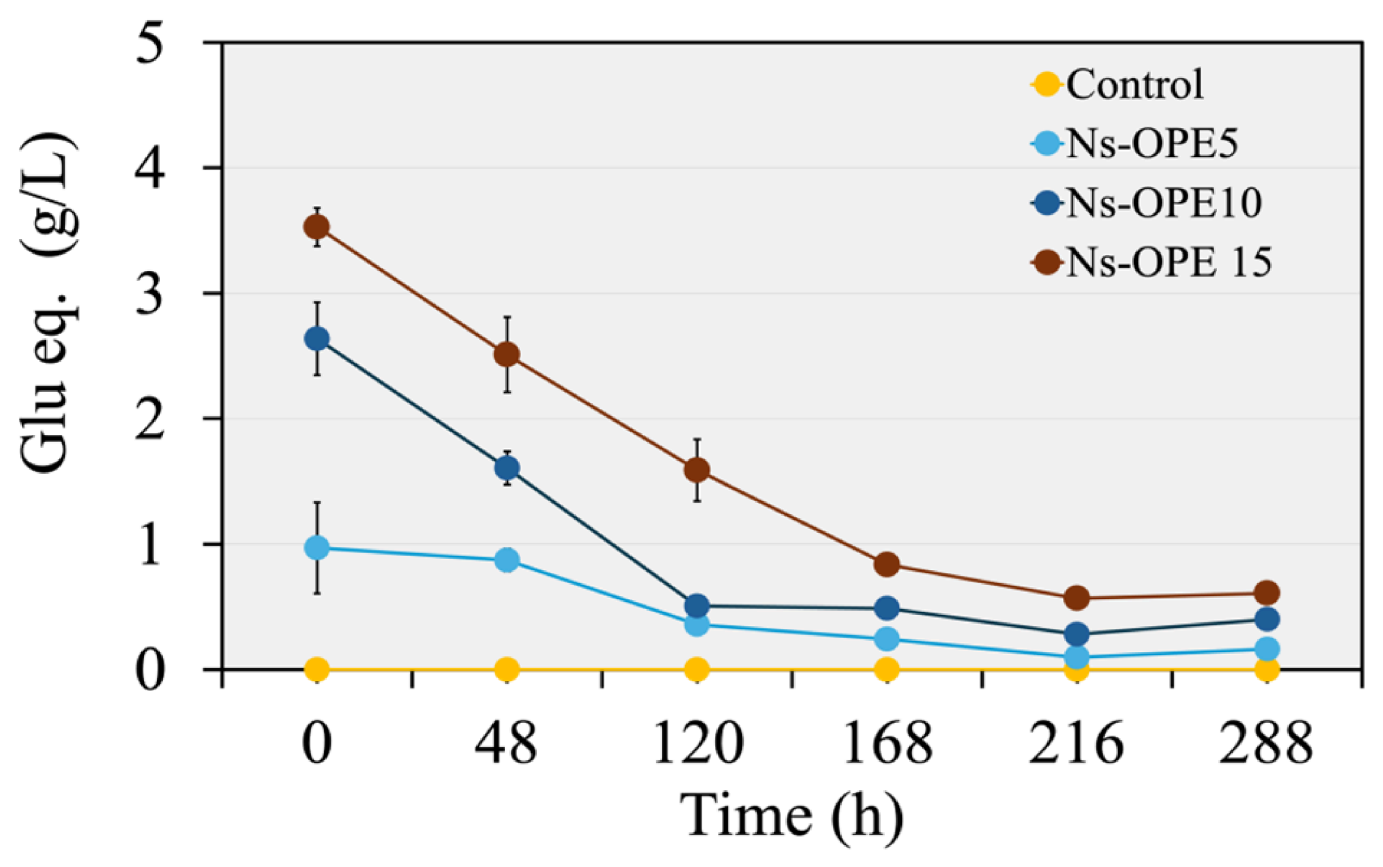
Variation in reducing sugars (glucose equivalent mg/mL) (0–288 h) in cultures of *N. salina* with different OPE concentrations. Values are means ± SD, *n* = 3.

The quantification of residual sugars in the medium (Figure 4) shows that, after 216 h, their levels decrease to below 15% in all tested conditions. In mixotrophic cultures, the supply of organic carbon can sustain growth even under self-shading or at high cell density, and several studies have reported that glucose supplementation may increase growth rates by 2–3-fold [44,45,46,47]. However, the addition of OPE, particularly at higher concentrations, may impose physiological constraints associated with altered nutrient availability and increased organic supplementation. Under this condition, the sugars present in the medium appear to be used predominantly for cellular maintenance rather than supporting increased biomass accumulation, contrary to what is typically observed in mixotrophic cultures [44,45,46,47]. This interpretation is consistent with the stable OD values observed across all treatments, even under nitrogen-limited conditions, suggesting that cells maintain metabolic activity without substantial increases in cell number.

**Figure 5:**
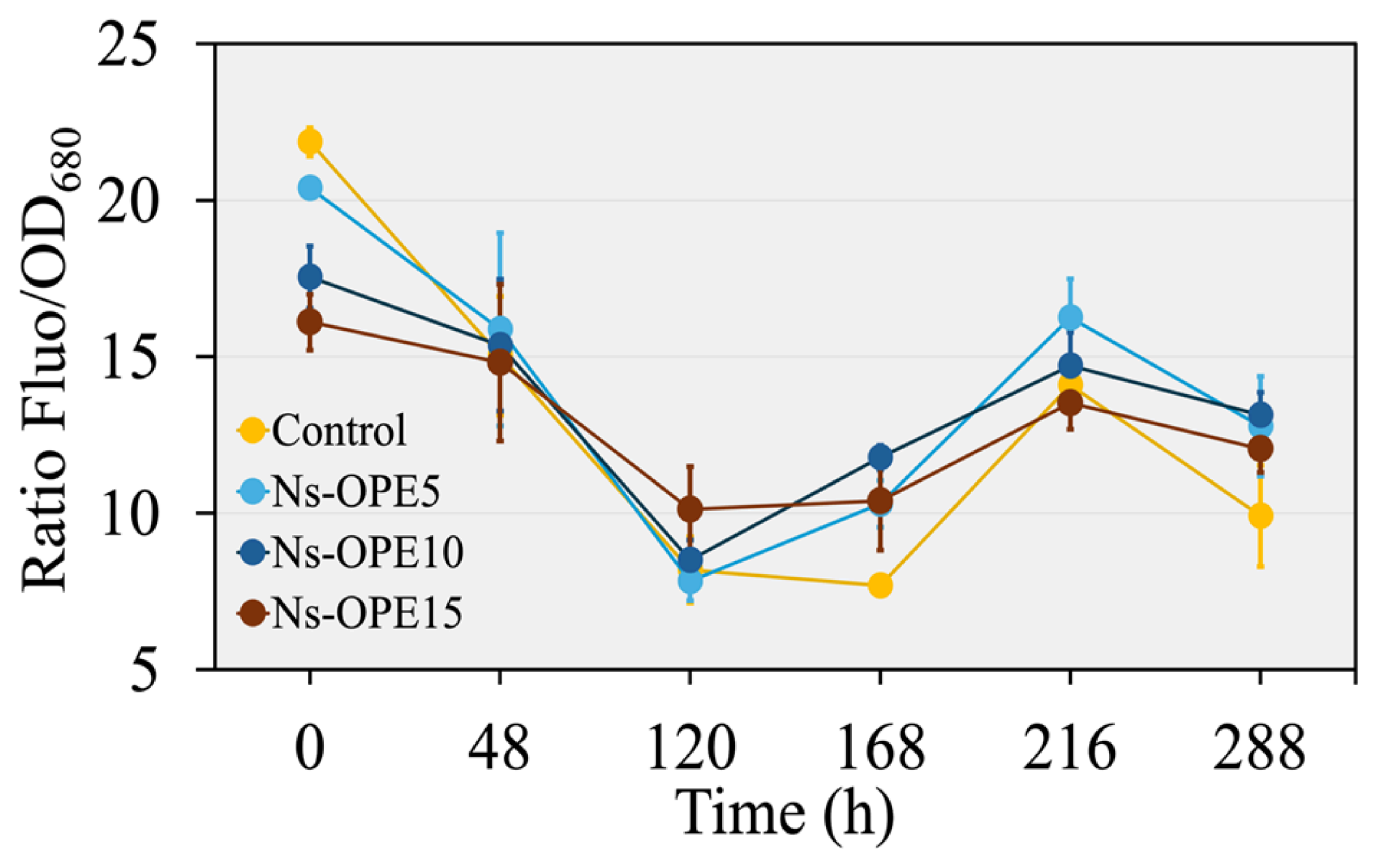
Ratio between chlorophyll fluorescence and OD_680_ over time in *N. salina* cultures grown with different OPE concentrations. Values are means ± SD, *n* = 3.

In our study, the ratio between fluorescence and OD_680_ (Figure 5), used as an indicator of chlorophyll relative to biomass, exhibited an initial decline during the first 120 h, followed by an increase up to 216 h. This trend could be indicative of an acclimation response of the photosynthetic apparatus associated with the progressive reduction of nitrogen availability in the culture medium. Similar dynamics under nitrogen stress conditions have been previously described [31].

During this intermediate phase, the increase in the fluorescence/OD ratio could reflect a relative increase in chlorophyll content compared to biomass, consistent with photo acclimatization processes reported for microalgae subjected to moderate stress or decreased growth rates. Subsequently, beyond 216 h, the new decrease in the ratio could be compatible with the onset of a more marked nitrogen limitation, as also observed in other species of the genus *Nannochloropsis* [6].

### 3.4. Modulation of Lipid Profiles in N. salina

**Figure 6:**
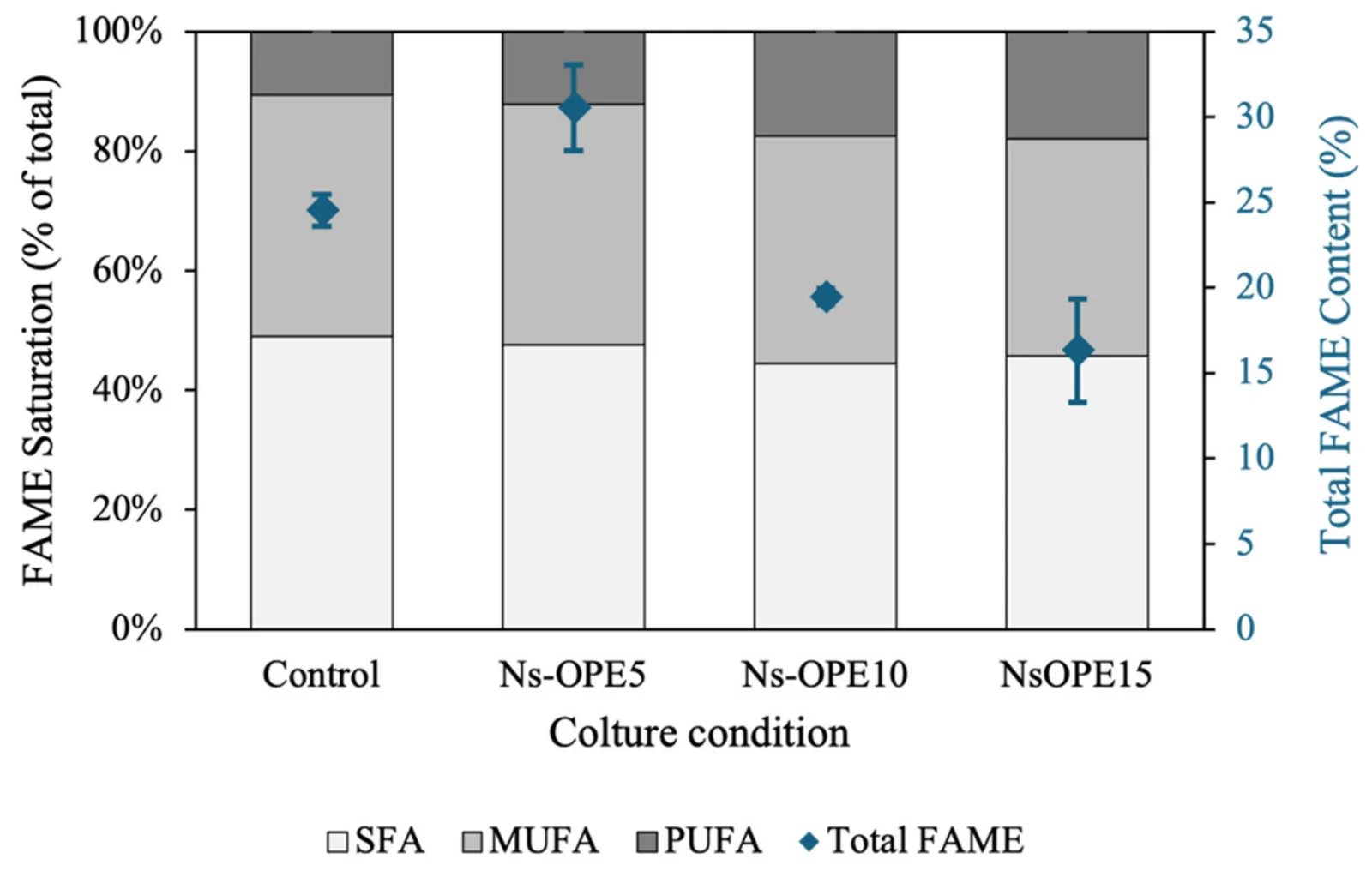
Comparison of fatty acid saturation profiles in *N. salina* cultivated with different OPE percentages. Polyunsaturated (PUFA), monounsaturated (MUFA), and saturated fatty acids (SFAs) are shown on the left *y*-axis, while total fatty acid content (% of dry weight) is reported on the right *y*-axis.

The fatty acid composition of microalgae is highly variable and mainly includes saturated fatty acids (SFAs), monounsaturated fatty acids (MUFAs), and PUFAs. Their biological roles depend on the type; SFAs contribute to membrane stability, while MUFAs and PUFAs confer membrane fluidity and dynamic functionality. The relative distribution of these lipid classes can vary significantly in response to growth conditions and different environmental stress factors, such as nutrient availability, light, or temperature, affecting both the nutritional quality and the functional properties of microalgal lipids [38,44]. As shown in Figure 6, the fatty acid (FA) profile was dominated by SFAs (45–49%) and MUFAs (36–40%), with lower proportions of polyunsaturated fatty acids (PUFAs, 10–18%). The total fatty acid content of the dry biomass varied among treatments. The control culture showed a total fatty acid content of 24.5 ± 0.9%, while cultures supplemented with OPE had values of 30.6 ± 2.5% (OPE5), 19.5 ± 0.4% (OPE10), and 16.3 ± 2.9% (OPE15). However, when lipid accumulation is evaluated on a volumetric basis (Table S2), a clear limitation emerges. Although OPE supplementation promoted changes in fatty acid composition and increased the relative abundance of PUFAs and EPA at the cellular level, these effects were offset by a concomitant reduction in biomass concentration. As a result, overall volumetric lipid and EPA productivities declined with increasing OPE levels. This outcome highlights a trade-off between improving lipid quality and maintaining culture performance, suggesting that, under the conditions tested, OPE addition is unlikely to be a suitable strategy for large-scale cultivation processes, where volumetric productivity is a critical factor.

The fatty acid profile of *N. salina* grown under photoautotrophic and mixotrophic conditions is reported in Table 3. Marked differences in lipid composition were also observed among treatments. Palmitic acid (C16:0) was the predominant fatty acid in all cultures (32–38%), followed by palmitoleic acid (C16:1), which accounted for 27–31%. Minor but consistently detected components included oleic acid (C18:1, 5–11%) and eicosapentaenoic acid (EPA, C20:5 n-3, 6–10%), along with traces of myristic acid (C14:0), stearic acid (C18:0), linoleic acid (C18:2), α-linolenic acid (C18:3 n-3), γ-linolenic acid (C18:3 n-6), and arachidonic acid (C20:4 n-6). The lipid composition of the control culture is consistent with previous reports for *N. salina* [40,48]. Cultures grown with OPE exhibited pronounced alterations in their fatty acid profile. Specifically, decreases in C16:0 and C16:1 were observed together with increases in C18:1, C18:2, and C20:5 n-3, with the largest changes occurring in OPE10 and OPE15. Lipid metabolism in *Nannochloropsis* is strongly influenced by culture conditions, including light regime [46], physiological state, and nutrient availability [40,49,50,51]. Although nitrogen did not decrease in absolute terms, OPE supplementation increased phosphorus levels, resulting in a marked decrease in the N:P ratio. Since many physiological responses attributed to nitrogen deficiency depend on the balance between N and P rather than on absolute N content, an imbalance towards phosphorus can alter lipid accumulation, as reported in *N. salina* [31,48]. Our results show a reduction in total fatty acids with increasing OPE concentration, except for OPE5, which exhibited a 6% increase relative to the control. This may indicate that the N:P ratio in the higher OPE treatments imposes restrictive conditions that prevent the typical lipid accumulation observed under nitrogen deficiency, whereas OPE5 maintains a more favorable balance that allows lipid enhancement. Interestingly, cultures exposed to the highest OPE concentrations displayed an average increase of approximately 3% in EPA relative to the control, consistent with a broader shift toward higher MUFA and PUFA proportions. The redistribution of lipid classes in OPE-treated cultures is also evident when examining absolute fatty acid contents (Table S3). In the OPE5 condition, absolute levels of C18:1, C18:2, and C20:5 n-3 were all elevated relative to the control. In particular, EPA increased by an average of 33% (mg g^−1^) in OPE5, whereas its level in OPE15 was comparable to the control. The literature indicates that reductions in EPA (20:5 n-3) fatty acids under nitrogen deprivation [31] are typically associated with declines in monogalactosyldiacylglycerol (MGDG) and digalactosyldiacylglycerol (DGDG), the major thylakoid membrane lipids. However, no reduction, either in relative or absolute terms, was observed in our experiments. Moreover, absolute EPA content (mg/g) correlated positively with extracted chlorophyll (Table S1; Pearson’s correlation coefficient (r) = 0.610, *p* < 0.05), suggesting that chloroplast integrity was not impaired by OPE supplementation and that the observed lipid changes arise from metabolic reallocation rather than structural membrane degradation.

Although the EPA content of *N. salina* reported in our analysis is relatively modest (approximately 2.07 g of EPA per 100 g of dry biomass), this value remains attractive when compared to traditional EPA sources such as cod liver oil (6.9 g/100 g), salmon oil (13 g/100 g), and sardine oil (10.1 g/100 g) [5] and in light of the sustainable and scalable cultivation of microalgae, especially considering the environmental impact of fish harvesting. Moreover, it should be emphasized that *N. salina* lipid extracts contain EPA predominantly as polar lipids (glycolipids and phospholipids), which ensure higher bioavailability than triacylglycerol-based oils, making them particularly suitable for nutritional applications [52].

### 3.5. Phenolic Content and Antioxidant Capacity

The total antioxidant capacity (TAC) increased by approximately 15–45% at low and intermediate OPE levels, reaching a maximum increase of 45% (*p* < 0.01) in cultures treated with 10% OPE. However, TAC decreased at the highest OPE concentration. Total phenolic content (TPC) across the different conditions remained comparable to the control (Table 4), indicating that the accumulation of phenolic compounds per unit of biomass was not significantly affected by the presence of OPE. As with other metabolites, volumetric productivity of TAC and TPC was largely limited by the reduced biomass in OPE-supplemented cultures, highlighting the importance of considering both cellular content and biomass accumulation.

In this context, the stability of the normalized TPC on biomass suggests that OPE primarily influenced growth, rather than the intrinsic capacity of *N. salina* to synthesize phenolic compounds. These results highlight the value of using OPE as a functional supplement, as its bioactive compounds, including flavonoids, phenols, and sugars, can stimulate the cellular antioxidant system, improving TAC without compromising the phenolic composition of the biomass, even under stressful conditions. Several phenolic compounds have been identified in *N. salina* that may contribute to its antioxidant activity, including gallic acid, 3,4-dihydroxybenzoic acid, ferulic acid (the most abundant), p-coumaric acid, and salicylic acid [53]. Although direct associations between antioxidant activity and specific metabolites remain limited, correlations have been reported between TAC and various compound classes, including phenolics, flavonoids, carotenoids, PUFAs, and protein/peptide fractions [41]. In our study, Pearson’s correlation analysis (Table S1) revealed strong positive relationships between chlorophyll content and both TAC (r = 0.851; *p* < 0.001) and TPC (r = 0.882; *p* < 0.001). Additionally, TPC correlated positively with total carotenoids (r = 0.881; *p* < 0.001) and TAC (r = 0.659; *p* < 0.05). These results are consistent with observations in other *Nannochloropsis* species [54] and support the observation that, although phenolics and carotenoids belong to distinct metabolic classes, they share a common functional role in the cellular antioxidant response.

From a pharmaceutical development perspective, the results highlight the feasibility of selectively modulating the antioxidant response of *N. salina* through natural supplements such as OPE. The combination of an enhanced antioxidant response and a stable phenolic profile suggests that the biochemical quality of the biomass is maintained, despite the reduction in productivity. The combined presence of phenolic compounds, carotenoids, and PUFAs, which have been associated with antioxidant and other bioactive properties in previous studies, suggests a potential value of *N. salina* biomass for future nutraceutical and biomedical applications. However, specific bioactivities and the contribution of individual metabolites require further validation. Moreover, the observation that these metabolites increase under controlled stress conditions suggests possible value in therapeutic strategies that rely on cellular stress modulation. Overall, these findings support the potential of *N. salina* as a promising source of high-value bioactive extracts, with possible applications in the nutraceutical sector and as a candidate for future developments within the marine drug field. However, further metabolomic characterization and targeted bioactivity assays are required to confirm specific applications.

### 3.6. Biomass Characterization by FT-IR Spectroscopy

The carbon source plays a pivotal role in microalgal growth and metabolic regulation. Carbon is required for nearly all cellular biosynthetic pathways, and its intracellular allocation drives substantial shifts in the biochemical composition of the biomass. Consequently, both the type and the concentration of the carbon source represent critical parameters in culture design, particularly when the objective is to modulate the synthesis of specific metabolites or to induce controlled carbon-stress responses.

**Figure 7:**
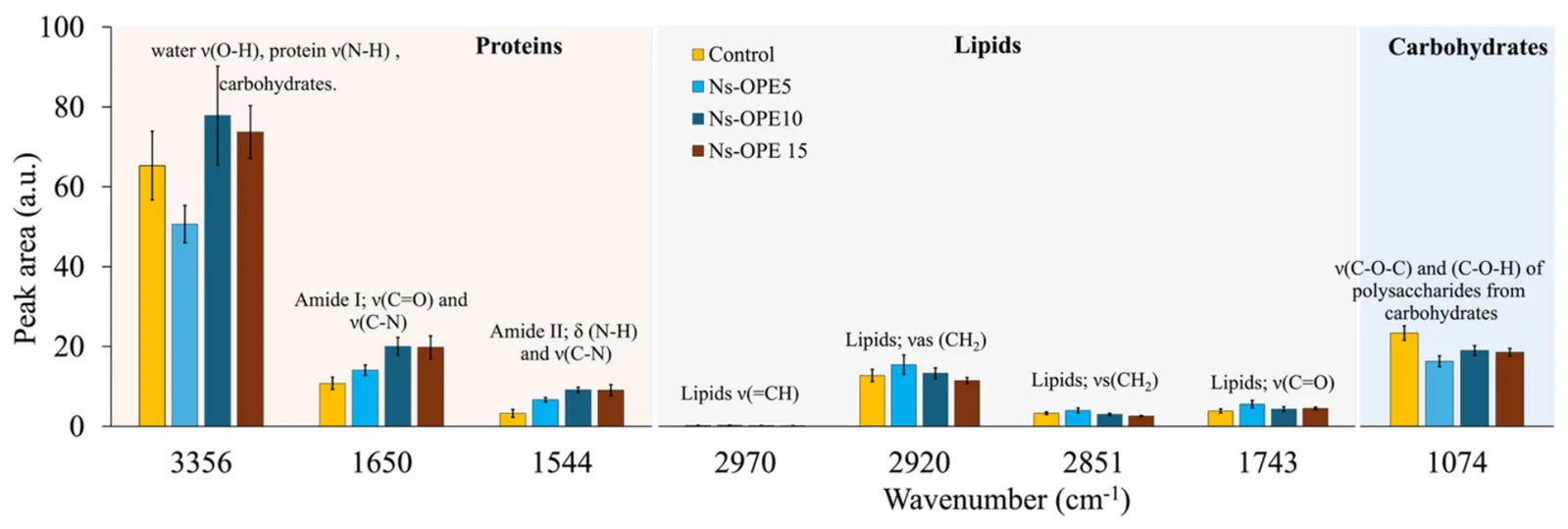
Peak areas of bands at specific wavenumbers from FT-IR spectra derived from *N. salina* in autotrophy and mixotrophy with different OPE concentrations after 288 h. Areas were determined from spectra reported in Figure S1. The area value of each band is the mean ± SD, *n* = 3.

Biochemical variations in *N. salina* exposed to different OPE concentrations were evaluated through comparative FT-IR analysis (Figure S1 and Table S2). The resulting spectral differences, summarized in Figure 7, highlight changes in the major structural components of the microalgal biomass—lipids, proteins, and carbohydrates. These molecular classes play essential and distinct roles in cellular physiology: lipids function not only as energy reserves but also as key constituents of biological membranes; proteins contribute to structural organization and catalyze the enzymatic reactions that regulate cellular responses to environmental cues; carbohydrates serve as storage compounds and participate in the formation of intra- and extracellular structural matrices. The observed spectral variations therefore reflect the physiological adjustments adopted by the microalga in response to increasing OPE concentrations.

Carbohydrates, represented by the band at 1074 cm^−1^, show a marked decrease in the presence of OPE, dropping from 30% in the control to 19% in the OPE 5% culture. In contrast, the protein-associated bands—Amide I (1650 cm^−1^) and Amide II (1544 cm^−1^)—exhibit substantial increases with rising OPE concentration, corresponding to approximately 30–80% and 100–180% increases relative to the control, respectively.

The lipid-related bands at 2920 cm^−1^, 2851 cm^−1^, and 1743 cm^−1^, assigned to fatty-acid chains and ester carbonyl groups, display a slight increase in the *Ns*-OPE5 culture compared with both the control and the higher OPE treatments. This trend is consistent with the total FAME content determined by GC analysis (Figure 6, Table S3).

Minor variations were also detected in the 900–1100 cm^−1^ region, which may correspond to phosphorylated biomolecules—including inorganic polyphosphates—and could qualitatively reflect an OPE-induced reorganization of phosphorus-containing compounds. Alternatively, these changes may be associated with carboxylated carbohydrate signals arising from extracellular polysaccharide substances produced as part of the metabolic adjustments occurring under mixotrophic conditions.

Similar trends have been observed in other studies on *Nannochloropsis* sp. Mitra and Mishra [55] reported that the presence of glucose led to changes in algal biomass composition, particularly in lipids and proteins. In their study, lipid content increased in the presence of glucose and protein content also increased at glucose concentrations of 2.5 g L^−1^, which are comparable to the levels estimated in our extracts. Pearson’s correlation analysis (Table S1) highlights several physiologically relevant relationships. The pH correlates negatively with Amide I (r = −0.834; *p* < 0.001) and Amide II (r = −0.928; *p* < 0.001) and positively with carbohydrates (r = 0.728; *p* < 0.01). Amide I also correlates positively with EPA content (r = 0.752; *p* < 0.01). Amide II correlates positively with TAC (r = 0.651; *p* < 0.05) and with EPA (r = 0.746; *p* < 0.005). Interestingly, these correlations suggest that variations in protein-associated signals may be associated with changes in lipid metabolism and membrane organization in *N. salina*. Because EPA is mainly incorporated into membrane glycerolipids, changes in EPA accumulation may be associated with broader cellular remodeling processes involving membrane composition and metabolism. Therefore, the positive correlation between EPA and protein-associated signals such as Amide II should be interpreted as reflecting coordinated physiological responses rather than a direct relationship between fatty acid accumulation and protein content.

## 4. Conclusions

The present study identified growth conditions that favor an increase in EPA content in *N. salina* oil, particularly when OPE was supplied at 5% as a supplement. Under these conditions, TAC was also enhanced, and a general trend of increasing polyunsaturated fatty acids was observed with rising OPE concentrations. Importantly, OPE supplementation did not induce major alterations in the primary metabolic profile of the biomass, suggesting that the observed metabolic changes occurred without extensive reprogramming of core cellular metabolism. Although OPE did not significantly increase biomass production, it represents a promising strategy to positively modulate the biochemical composition of microalgal biomass, particularly by enhancing polyunsaturated fatty acid content. These findings further support *N. salina* as a suitable marine microalgal strain to produce high-value bioactive compounds of nutraceutical and pharmaceutical interest.

## Figures and Tables

**Table 1:** Culture media for the growth of *N. salina* with different OPE concentrations.

Medium	Culture	N-NO_3_ (mg/L)	P-PO_4_^3−^ (mg/L)	N:P (Molar Ratio)
f/2	Control	12.4	1.3	21.1
f/2 + 5% OPE	*Ns*-OPE5	12.4	3.5	5.7
f/2 + 10% OPE	*Ns*-OPE10	12.4	7.0	3.3
f/2 + 15% OPE	*Ns*-OPE15	12.4	10.5	2.3

**Table 2:** Dry weight of cultures, chlorophyll and total carotenoids after 288 h. Cultures of *N. salina* (Ns) at different OPE concentrations (5%, 10% and 15%). Values are means ± SD, *n* = 3.

Culture	Dry Biomass (g/L)	Chl *a* (mg/g)	TC * (mg/g)
Control	0.246 ± 0.016	17.50 ± 1.75	8.24 ± 0.61
*Ns*-OPE5	0.171 ± 0.010 ^c^	20.25 ± 2.05	8.26 ± 1.39
*Ns*-OPE10	0.172 ± 0.007 ^c^	22.64 ± 1.28 ^a^	8.75 ± 0.38
*Ns*-OPE15	0.176 ± 0.014 ^c^	16.67 ± 1.58	7.09 ± 0.58

* Total carotenoids; a and c indicate significant differences at *p* < 0.05 and *p* < 0.001, respectively, compared with the control culture.

**Table 3:** Fatty acid composition of *N. salina* grown in f/2 medium with different concentrations of OPE. The table shows the relative percentage (mean ± SD, *n* = 3) of total fatty acids.

Categories	Culture	Control	*Ns*-OPE5	*Ns*-OPE10	*Ns*-OPE15
SFA	C14:0	4.8 ± 0.2	4.8 ± 0.2	5.6 ± 0.3 ^a^	5.3 ± 0.2
SFA	C16:0	38.1 ± 1.4	37.4 ± 0.8	32.7 ± 1.8 ^a^	32.2 ± 3.2 ^a^
SFA	C18:0	4.3 ± 0.6	3.1 ± 0.1 ^a^	3.7 ± 0.4	4.4 ± 0.4
MUFA	C16:1	31.6 ± 0.4	31.0 ± 1.2	30.1 ± 0.5	27.8 ± 1.4 ^b^
MUFA	C18:1	8.4 ± 0.2	11.0 ± 1.2 ^a^	6.2 ± 0.4	5.5 ± 1.3 ^b^
PUFA	C18:2	2.1 ± 0.2	2.9 ± 0.1	4.3 ± 0.3 ^c^	5.1 ± 0.6 ^c^
PUFA	C18:3 n3	0.5 ±0.2	0.5 ± 0.0	0.4 ± 0.0 ^c^	0.3 ± 0.1 ^c^
PUFA	C18:3 n6	0.0 ± 0.0	0.3 ± 0.1	0.8 ± 0.2	1.1 ± 0.2 ^a^
PUFA (ARA)	C20:4 n6	1.0 ± 0.2	1.1 ± 0.1	1.4 ± 0.2	1.3 ± 0.1
PUFA (EPA)	C 20:5 n3	6.4 ± 1.1	6.8 ± 0.5	9.8 ± 1.7 ^a^	9.3 ± 1.8
SFA	Others	2.3 ± 0.8	2.8 ± 1.1	3.0 ± 1.2	4.4 ± 2.1

a, b, c indicate significant differences at *p* < 0.05, *p* < 0.01, and *p* < 0.001, respectively, compared with the control culture.

**Table 4:** Antioxidant capacity: DPPH free radical scavenging assay (TAC) and Folin–Ciocalteu assay (TPC).

Culture	TAC (Vitamin C mg/g)	TAC (Vitamin C g/L)	TPC (mg GAE/g) *	TPC (mg GAE/L)
Control	4.6 ± 0.4	1.1 ± 0.1	8.9 ± 0.9	2.46 ± 0.33
*Ns*-OPE5	6.1 ± 0.4	1.0 ± 0.1	9.0 ± 1.0	1.72 ± 0.19
*Ns*-OPE10	6.7 ± 0.4 ^a^	1.2 ± 0.1	10.5 ± 0.9	2.02 ± 0.25
*Ns*-OPE15	5.3 ± 0.9	0.9 ± 0.1	7.6 ± 0.6	1.48 ± 0.11

* GAE: Gallic Acid Equivalent; Values are means ± SD (*n* = 3); a, indicates a significant difference at *p* < 0.05 compared with the control culture.

## Data Availability

Data are contained within the article. Further inquiries can be directed to the corresponding author.

## Supplementary Materials

The following supporting information can be downloaded at: https://www.mdpi.com/article/10.3390/biotech15030065/s1, Table S1. Influence of Orange Peel Extract (OPE) concentration on physiological and biochemical parameters of *N. salina*: A Correlation Matrix Approach; Table S2. Fatty acid of *N. salina* grown in f/2 medium with different concentration of OPE. The table shows the volumetric production of fatty acid in mg/L/day (mean ± SD, *n* = 3); Table S3. Fatty acid of *N. salina* grown in f/2 medium with different concentration of OPE. The table shows the content of fatty acid in mg per g of dry weight biomass (mean ± SD, *n* = 3); Table S4. Band assignments at different wavenumbers of FT-IR spectra of dry samples of microalgae biomass, as determined by Xu et al., 2024 [56]; Figure S1. Overlay of FTIR spectra (980–3900 cm^−1^) of *N. salina* grown under increasing OPE concentrations.

## Abbreviations

The following abbreviations are used in this manuscript:

OPE	Orange Peel Extract
CO_2_	Carbon Dioxide
EPA	Eicosapentaenoic Acid
PUFA	Polyunsaturated Fatty Acid
SFA	Saturated Fatty Acid
MUFA	Monounsaturated Fatty Acid
TAC	Total Antioxidant Capacity
TPC	Total Phenolic Content
Ns	*Nannochloropsis salina*
DNS	3,5-dinitrosalicylic acid
FAME	Fatty Acid Methyl Ester
