# Supplementary material for: Effects of Orange Peel-Derived Carbon Sources on Nannochloropsis salina Mixotrophic Cultivation
Source: BioTech (Basel). 2026 Aug 7;15(3):65. doi: 10.3390/biotech15030065 (PMC13511695; doi:10.3390/biotech15030065)
Supplement: Supplementary file 1 [file biotech-15-00065-s001.zip › biotech-4445216-supplementary.pdf]

BioTech

MDPI

Supplementary Materials: Effects of Orange Peel-Derived Carbon Sources on *Nannochloropsis salina* mixotrophic cultivation

Carlo Esposito, Giancarlo Aldini, Yanan Yin, Stefano Gandolfi, Gianluca Ottolina, Francesco Secundo

List of Tables

- **Table S1.** Influence of orange peel extract (OPE) concentration on physiological and biochemical parameters of *N. salina*: A Correlation Matrix Approach
- **Table S2.** Fatty acids of *N. salina* grown in f/2 medium with different concentrations of OPE. The table shows the volumetric production of fatty acids in mg/L/day (mean ± SD, n = 3).
- **Table S3**. Fatty acids of *N. salina* grown in f/2 medium with different concentrations of OPE. The table shows the volumetric production in mg of fatty acid per g of dry weight biomass. (mean ± SD, n = 3)
- **Table S4.** Selected band assignments at different wavenumbers of FTIR spectra as determined by (Xu et al., 2024). The letter "ν" denotes stretching vibrations; the letter "δ" denotes bending vibrations; the letter "s" denotes symmetric stretching or bending; the letter "as" denotes asymmetric stretching or bending.

List of Figures

- **Figure S1.** Overlay of FTIR spectra (900–3900 cm^{-1}) of *N. salina* grown under increasing OPE concentrations.

Table S1. Influence of orange peel extract (OPE) concentration on physiological and biochemical parameters of *N. salina*: A Correlation Matrix Approach.

	pH	OD_{680}	IF	DW(mg)	Chl *a* (µg/g)	TC (µg/g)	TAC	TPC	FA (%)	EPA (%)	Amide I	Amide II	Carboidrates	EPA(mg/g)
pH	1													
OD_{680}	0.718**	1												
IF	- 0.375	-0.043	1											
DW(mg)	0.892***	0.665*	-0.569	1										
Chl *a* (µg/g)	- 0.247	- 0.253	0.402	- 0.436	1									
TC (µg/g)	0.200	- 0.072	0.119	0.028	0.815**	1								
TAC	- 0.561	- 0.420	0.340	- 0.660*	0.851***	0.531	1							
TPC	0.042	- 0.073	0.211	- 0.074	0.882***	0.881***	0.659*	1						
FA (%)	0.428	0.431	0.123	0.174	0.097	0.153	- 0.053	0.105	1					
EPA (%)	- 0.642*	- 0.517	0.147	- 0.557	0.338	0.039	0.561	0.111	-0.740**	1				
Amide I	- 0.834***	- 0.504	0.284	- 0.717**	0.262	- 0.148	0.558	0.077	- 0.668*	0.752**	1			
Amide II	- 0.928***	- 0.621*	0.338	- 0.857***	0.343	- 0.107	0.651*	0.081	- 0.551	0.746**	0.967***	1		
Carboidrates	0.728**	0.622*	- 0.448	0.768**	- 0.291	0.005	- 0.486	0.009	- 0.164	- 0.228	- 0.273	-0.843	1	
EPA(mg/g)	- 0.193	-0.001	0.448	-0.426	0.610*	0.294	0.617*	0.332	0.182	-0.034	0.125	-0.524	0.505	1

Note: IF: Fluorescent Intensity; TC: Total carotenoids; TAC: Total Antioxidant Content; TPC: Total phenolic content; FA: Fatty acid; EPA: eicosapentaenoic acid; * $p < .05$, ** $p <.01$, and ***$p <.001$.

 https://doi.org/10.3390/15030065

Table S2. Fatty acids of *N. salina* grown in f/2 medium with different concentrations of OPE. The table shows the volumetric production of fatty acids in mg/L/day (mean ± SD, n = 3).

Categories	Culture #	*Control*	*Ns-OPE5*	*Ns-OPE10*	*Ns-OPE15*
SFA	C14:0	0.243 ± 0.033	0.207 ± 0.010	0.155 ± 0.003[a]	0.128 ± 0.036[b]
SFA	C16:0	1.924 ± 0.257	1.625 ± 0.143	0.913 ± 0.088[b]	0.792 ± 0.277[c]
SFA	C17:0	0.080 ± 0.029	0.097 ± 0.052	0.068 ± 0.033	0.082 ± 0.026
SFA	C18:0	0.218 ± 0.050	0.136 ± 0.011[a]	0.104 ± 0.013[b]	0.105 ± 0.021[b]
MUFA	C16:1	1.595 ± 0.181	1.344 ± 0.081	0.838 ± 0.048[b]	0.679 ± 0.216[c]
MUFA	C18:1	0.442 ± 0.024	0.401 ± 0.054	0.228 ± 0.021[c]	0.208 ± 0.066 [c]
PUFA	C18:2	0.107 ± 0.008	0.125 ± 0.005	0.120 ± 0.015	0.121 ± 0.019
PUFA	C18:3 n3	0.027 ± 0.006	0.021 ± 0.001	0.011 ± 0.001[b]	0.008 ± 0.004[b]
PUFA	C18:3 n6	0.000 ± 0.000	0.014 ± 0.003[b]	0.022 ± 0.004[c]	0.029 ± 0.006[c]
PUFA	C20:4 n6 (ARA)	0.052 ± 0.008	0.049 ± 0.007	0.039 ± 0.004	0.031 ± 0.006[a]
PUFA	C 20:5 n3 (EPA)	0.318 ± 0.034	0.295 ± 0.032	0.273 ± 0.047	0.219 ± 0.031[a]
SFA	Others	0.036± 0.007	0.026± 0.002	0.016 ± 0.002	0.016 ± 0.005

*Significant values with a ($p < 0.05$), b ($p < 0.01$), or c ($p < 0.001$) compared with the control culture.

Table S3. Fatty acids of *N. salina* grown in f/2 medium with different concentrations of OPE. The table shows the content of fatty acids in mg per g of dry weight biomass (mean ± SD, n = 3).

Categories	Culture #	*Control*	*Ns-OPE5*	*Ns-OPE10*	*Ns-OPE15*
SFA	C14:0	11.8 ± 0.8	14.6 ± 0.7[a]	10.8 ± 0.4	8.6 ± 1.7[a]
SFA	C16:0	93.5 ± 6.9	114.5 ± 11.2	63.8 ± 4.7[a]	53.2 ± 14.4[b]
SFA	C17:0	3.9 ± 1.5	6.7 ± 3.5	4.7 ± 2.2	5.7 ± 2.3
SFA	C18:0	10.6 ± 1.8	9.6 ± 0.9	7.3 ± 0.8[a]	7.1 ± 1.0[a]
MUFA	C16:1	77.5 ± 3.9	94.8 ± 8.0	58.6 ± 1.6[a]	45.7 ± 10.7[b]
MUFA	C18:1	21.6 ± 0.5	28.2 ± 3.2[c]	15.9 ± 1.1[c]	14.0 ± 3.6[c]
PUFA	C18:2	5.2 ± 0.3	8.8 ± 0.5[c]	8.3 ± 0.7[c]	8.2 ± 0.6[c]
PUFA	C18:3 n3	1.3 ± 0.3	1.5 ± 0.1	0.8 ± 0.0[c]	0.5 ± 0.2[c]
PUFA	C18:3 n6	0.0 ± 0.0	1.0 ± 0.2	1.6 ± 0.3[a]	2.0 ± 0.3[b]
PUFA	C20:4 n6 (ARA)	2.6 ± 0.5	3.4 ± 0.3	2.7 ± 0.3	2.1 ± 0.2
PUFA	C 20:5 n3 (EPA)	15.5 ± 2.0	20.7 ± 1.3[a]	19.1 ± 3.0	14.9 ± 1.3
SFA	Others	1.8 ± 0.2	1.9 ± 0.2	1.2 ± 0.1	1.1 ± 0.2

*Significant values with a ($p < 0.05$), b ($p < 0.01$), and c ($p < 0.001$) compared with the control culture.

Table S4. Band assignments at different wavenumbers of FT-IR spectra of dry samples of microalgae biomass, as determined by (Xu et al., 2024).

Wavenumber (cm^{-1})	Band Assignment[a]
3356	ν(O-H) of water and ν(N-H) of amides from proteins
2970	ν(=CH) of unsaturated lipids
2920	νas (CH2) of fatty acids from lipids
2851	νs(CH2) of fatty acids from lipids
1743	ν(C=O) of esters from lipids (TAG)
1650	ν(C=O) and ν(C-N) of amides from proteins (Amide I)
1544	δ (N-H) and ν(C-N) of amides from proteins (Amide II)
1074	ν(C-O-C) and (C-O-H) of polysaccharides from carbohydrates

[a]The letter "ν" denotes stretching vibrations; the letter "δ" denotes bending vibrations; the letter "s" denotes symmetric stretching or bending; the letter "as" denotes asymmetric stretching or bending.

Figure S1. Overlay of FTIR spectra (980–3900 cm^{-1}) of *N. salina* grown under increasing OPE concentrations.

https://doi.org/10.3390/15030065

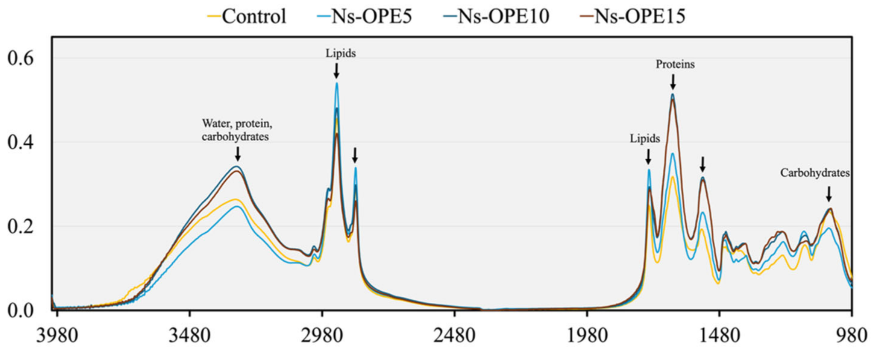

Control
Ns-OPE5
Ns-OPE10
Ns-OPE15
0.6
0.4
0.2
0.0
3980
3480
2980
2480
1980
1480
980
Lipids
Water, protein, carbohydrates
Lipids
Proteins
Carbohydrates
